# Standard v mini percutaneous nephrolithotomy in the supine modified lithotomy position: a randomized pilot study on 10–25 mm stones

**DOI:** 10.1111/ans.19227

**Published:** 2024-10-01

**Authors:** Philip McCahy, Anthony Dat, Daniel Gilbourd, Eldho Paul, Shekib Shahbaz

**Affiliations:** ^1^ Department of Urology, Monash Health Casey Hospital Melbourne Victoria Australia; ^2^ School of Clinical Sciences Monash University Melbourne Victoria Australia; ^3^ School of Public Health and Preventative Medicine Monash University Melbourne Victoria Australia

**Keywords:** complications, lithotripsy, percutaneous nephrolithotomy, renal stone, ureterorenoscopy

## Abstract

**Background:**

Percutaneous nephrolithotomy (PCNL) is the recommended treatment for stones >2 cm in size. The majority of PCNL are still conducted with larger telescopes using tracts up to 30F in size. We have conducted a randomized pilot study comparing mini PCNL with our standard 22F PCNL for renal stones between 10 and 25 mm in diameter.

**Methods:**

Patients were randomized to either PCNL (24F Amplatz sheath/22F nephrosocope) or mini PCNL (18F Amplatz sheath/11F nephroscope). All operations were performed in the modified supine position. Patients were reviewed with imaging to assess stone clearance and complications.

**Results:**

Eighteen well matched patients were randomized. All procedures were completed as planned and all were tubeless with no complications. There were no differences in operative time, analgesia requirements or length of stay. Seven of nine (77.75%) standard PCNL were completely stone free at CT review with a 2 mm and a 5 mm fragments in the other patients. Four (44.4%) of the mini PCNL group were stone free, with stone fragments 4–10 mm remaining in the others. 40 patients/arm would be required for an adequately powered study.

**Conclusion:**

There was no advantage in using mini PCNL compared to our standard 24F PCNL in this pilot study. There may be benefits in using mini PCNL compared to the more widely used 30F PCNL and it may be a more cost‐effective alternative to laser pyeloscopic stone procedures.

## Graphical abstract

PCNL using 24F tracts is as safe as mini PCNL with better stone clearance.

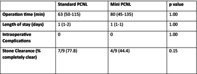

## Introduction

Percutaneous nephrolithotomy (PCNL) is the recommended treatment for renal stones over 2 cm.^1^ There is no consensus on the best treatment for renal stones sized between 10 and 20 mm but a number of studies have suggested that the best clearance rates are achieved with PCNL.^2^, ^3^ Mini PCNL, defined as a tract dilated from 11F to less than 20F, is a well‐established technique that has been advocated as a less invasive method of clearing stones compared to the standard operation^4^ though there have been few randomized studies comparing the techniques.

We set out to explore whether one technique was better than the other in a randomized pilot study for renal stones sized between 10 and 25 mm.

## Methods

All patients presenting to the authors with renal stones on a renal tract 2 mm slice CT scan (CT KUB) sized between 10 and 25 mm were offered access to the trial. If they agreed to participate, they were randomized to either standard PCNL or mini PCNL. A computer‐generated list of random treatment allocations was used to assign patients to treatment in a 1:1 ratio with the lead author and unit secretary ensuring accuracy and maintaining the data. There was no blinding. Data was collected prospectively and included patient demographics; stone position, size and CT density (measured in Hounsfield units); operative details including length of procedure, lithotripsy energy, intra‐operative complications; post‐operative length of stay, post‐operative complications and analgesia requirements; and post‐operative review with imaging (CT scans with 3 mm slices).

All procedures were performed in the modified supine position as previously described.^5^ The standard PCNL involved tract dilatation using a 24F balloon and Amplatz sheath (Boston Scientific, USA) and a 22F nephroscope (Olympus, Japan). The mini‐PCNL used an 11F nephroscope (Olympus, Japan) through an 18F Amplatz sheath after balloon dilatation (Cook Medical, USA). Various energy sources were available for lithotripsy including ultrasound, percussion (Lithoclast, EMS, Switzerland), 120W Holmium‐YAG laser (Lumenis, Israel) and thulium pulsed laser (Soltive, Olympus, Japan). All patients were given prophylactic antibiotics. Nephrostomy was only placed if there was bleeding or elevated risk of infection. No haemostatic agents were used in tract closure.

Statistical analysis was performed using SAS software version 9.4 (SAS Institute, Cary, NC, USA). Comparisons between groups (standard PCNL versus mini PCNL) were made using Wilcoxon rank‐sum test for continuous variables and chi‐square or Fisher's exact test as appropriate for categorical variables. Statistical significance was set at a two‐sided *P*‐value of 0.05.

The study was approved by the local Human Research and Ethics Committee (HREC/17/MonH/226).

## Results

**Fig. 1:**
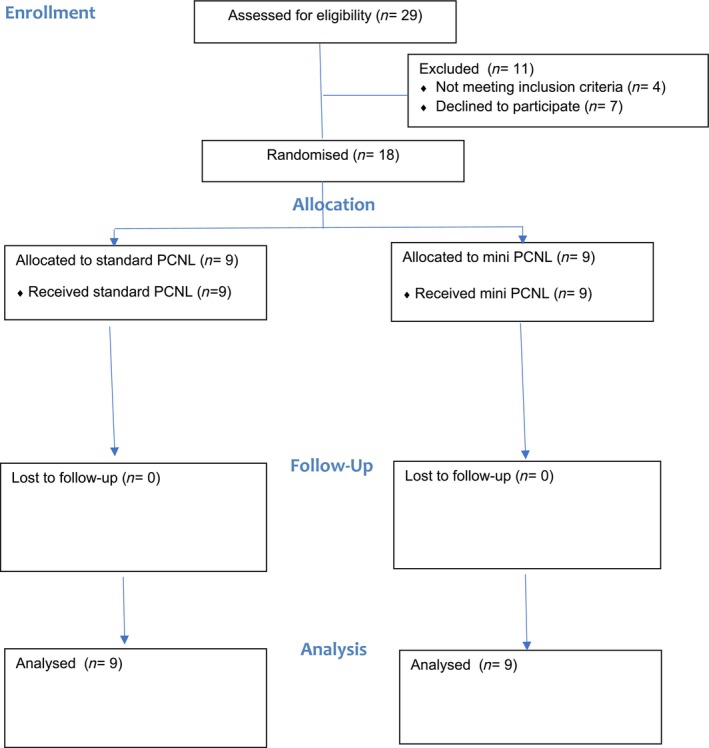
Consort flow chart.

A total of 18 patients were recruited with nine allocated to each of the study arms. The Consort Flow Chart (Fig. 1) details how the study was conducted and patient demographics are shown in Table 1. The patients were well matched for age, stone size and density, and Body Mass Index. Operation details are shown in Table 2 with each procedure completed as planned. No patient underwent ureterorenoscopy (URS).

**Table 1:** Patient demographics

	Standard PCNL	Mini PCNL	*P*‐value
Age	54 (25–68)	56 (35–71)	1.00
Sex (M:F)	5:4	4:5	1.00
BMI	29 (19.8–37)	29.4 (22.7–36.8)	1.00
Stone size (mm)	15 (10–19)	16 (13–20)	1.00
Hounsfield Units	1269 (423–1634)	1217 (606–1563)	1.00
Skin to stone distance (mm)	91 (84–130)	94 (77–113)	1.00

**Table 2:** Operation and post‐operative details

	Standard PCNL	Mini PCNL	*P*‐value
Operation time (min)	63 (50–115)	80 (45–135)	1.00
Length of stay (days)	1 (1–2)	1 (1–1)	1.00
Intraoperative complications	0	0	1.00
Stone clearance (% completely clear)	7/9 (77.8)	4/9 (44.4)	0.15

There were no intraoperative complications and no post‐operative blood transfusions. All procedures were able to be done completely tubeless and all but one of the patients (who underwent standard PCNL and stent removal and stayed in hospital two nights) were discharged home on the first post‐operative day. One patient from the mini‐PCNL group represented at post‐op day 9 with clot colic and was stented as an emergency.

Post‐operative analgesic requirements were similar between the two groups. Two patients in the mini‐PCNL group only required oral paracetamol during their stay. The remainder were given either low dose parenteral opioids (tramadol or fentanyl) or oxycodone in accordance with a standard post‐operative pain pathway. One patient in the standard PCNL group had no post‐op analgesia at all and the remainder all had either low dose parenteral or oral opioids with additional oral paracetamol or non‐steroidal anti‐inflammatories.

All patients underwent out‐patient review with post‐operative imaging with both CT KUB and plain x‐rays within 6 months of the procedure. Whilst not statistically significant (Table 2) there was a trend towards more residual stones following mini‐PCNL. Only one of the five residual stones in the mini‐was less than 4 mm in diameter with the other four ranging in size between 5 and 10 mm. Two of these patients needed subsequent additional procedures – one extracorporeal shock wave lithotripsy and the other undergoing URS. Of the two patients in the standard PCNL groups who were not completely stone free the residual stones were sized 2 mm and 5 mm. No additional procedures were performed in this group.

This pilot study has enabled us to calculate that 40 patients per treatment arm are required to show a 33% difference in stone clearance with 80% power at 5% level of significance.

## Discussion

The options for treating renal stones have been very dependent on technological advances. The increasing sophistication of URS and laser developments have allowed for a massive increase in the use of retrograde approaches though there is increasing evidence that stone clearance may not be complete and the procedures are not complication free.^6^ PCNL is still the recommended treatment for larger stones.^2^ Mini PCNL was developed as a cost‐effective alternative to URS^4^ and has been increasingly used to target larger renal stones.^7^

This pilot study has looked at stone clearance rates and analgesic use with mini‐PCNL and our standard PCNL for medium sized stones. We have found no difference in pain but a trend towards improved stone clearance with the larger puncture/nephroscope. We feel that this is because, particularly in the modified supine position, drainage of stone fragments is easier through the larger tract, visualization of the collecting system is better with the larger scope and flexible nephroscopes can be used to confirm clearance. A flexible ureterorenoscope can be inserted through the mini tract but the field of view and ability to remove stones is not as good as with the larger flexible scope. Retrograde URS could also be considered for all mini PCNL patients but this would almost certainly require ureteric stenting which would increase patient discomfort.

The CROES study suggested that the majority of PCNL worldwide are performed with 30F tracts and Amplatz sheaths^8^ and, anecdotally, that is still the case. The authors have not routinely used these larger tracts (and the associated larger nephroscopes) for over a decade. There has not been a study looking at the difference in outcomes using the different sized sheaths, but we postulate that the larger tracts cause more renal trauma and bleeding with little benefit for stone clearance. Mini PCNL compared to these larger tracts will probably prove advantageous but has little to offer compared to the 22F nephroscope/24F Amplatz that we use.

A previous studies looking at 24F tracts versus mini‐PCNL^8^ suggested more trauma and longer stays more in keeping with a reported meta‐analysis.^9^ The latter mostly compared mini‐PCNL with the larger 30F tracts for stones of all sizes. The current study shows that PCNL and mini PCNL for smaller renal stones are equally safe and well tolerated. All of the procedures were performed with no nephrostomy or ureteric stents, that is, totally tubeless, and, with one exception, patients were able be discharged from hospital the day after surgery. We believe that in the absence of a strong indication (long operative time, intraoperative bleeding, and concern about sepsis) nephrostomy placement may be involved in ongoing trauma to the percutaneous tract and prolong or precipitate bleeding. These results are probably due to the minimally traumatic telescopes and renal access used as well as being a high‐volume unit having expert theatre and ward teams that are used to a rapid patient throughput with an expectation that patients will recover and mobilize quickly. The CROES study^8^ also looked at transfusion rates noting that larger tracts increased the risk of bleeding. We have not found that in this study though the 18F tract used for mini PCNL is at the top end of the sizing definitions.

Limited access to expensive URS equipment and high cost of repair of the delicate telescopes, may still allow for mini‐PCNL to be a cost‐effective option for larger stones in some units. The main advantage in mini‐PCNL may lie in treating smaller renal stones, in particular those now almost universally treated with URS. There are likely to be both better stone clearance and cost savings using mini‐PCNL though the lack of expertise in achieving renal access required for this technique may prevent widespread uptake. We are addressing these issues in another study comparing URS with mini PCNL.

Limitations of this study include the small numbers and the inability to blind the modality used. Due to the relatively small size of the stones studied many suitable patients were not allocated for possible recruitment and, instead, booked for URS. Some of the enrolment phase was during the COVID‐19 pandemic when patients were not physically seen in clinics and only urgent operations (mostly for much larger stones) were performed. The aim of calculating numbers for an adequately powered definitive study was achieved.

## Author contributions

**P. McCahy:** Conceptualization; data curation; methodology; project administration; writing – original draft. **A. Dat:** Data curation; Edit text. D. Gilbourd Ethics application; Data curation; Edit text. E. Paul Statistical analysis. S. Shahbaz Clinical input. Edit text.

## Conflict of interest

The authors did not receive support from any organization for the submitted work. A/Prof McCahy has been a paid consultant for Boston Scientific. The other authors have no competing interests to declare that are relevant to the content of this article.
